# 

**Published:** 1921-12

**Authors:** 


### ANNOUNCEMENTS

### Annual Convention Texas State Dental Society

The Texas State Dental Society will hold its Forty-second Annual Convention, March 13 and 14, 1922, at Houston, Texas.

The special features of the program will be lecture and clinic work by Dr. Boyd S. Gardner, of Rochester, Minn., and Dr. Carl Hoffer, of Nashville, Tenn., with symposiums and unit clinics by members of the society.

During the four days following, a post-graduate class course will be conducted by Drs. Willis A. Coston, Topeka; Russell W. Tench, New York City; Arthur E. Smith,

Chicago; T. W. Maves, Minneapolis, and Julian Smith, Dallas.—J. G. Fife, *Secy.*, 1813 Main St., Dallas, Texas.

---

### American Institute of Dental Teachers

The Twenty-ninth Annual Meeting of the American Institute of Dental Teachers will be held at the Winsor Hotel, Montreal, Quebec, on January 23, 24, 25, 1922.

An interesting program has been arranged, built around the theme of Preventive Dentistry and its correlated subjects:—What, When, How, and Why to teach. There will also be a complete exhibit of scientific teaching apparatus, college equipment, etc.

All interested in dental teaching are cordially invited to attend.—Guy S. Millberry, *President;* Abram Hoffman, *Secretary-Treasurer;* 381 Linwood Ave., Buffalo, N. Y.

---

### Kentucky State Dental Association

"The next annual meeting of the Kentucky State Dental Association will be held in Louisville, Kentucky, April 10, 11, 12, 1922, Seelbach Hotel, headquarters." A clinical program of unusual interest is being arranged. Address all correspondence to W. M. Randall, secretary, Louisville, Kentucky. Yours fraternally,

W. M. Randall, *Secretary.*

---

### Electro-Radiographic Diagnosis

BY HOWARD R. RAPER, D.D.S.

This is a timely and remarkably well-expressed contribution on a subject little known and only written about incidentally as associated with other subjects. Dr. Raper has written a real book. It is not simply an essay on a favorite topic or a hobby, but it is written with a preface, table of contents of twelve chapters, and forty-three illustrations, all of which are admirably printed. This book has some things in it to admire and to study besides the things mentioned above that causes us to say that the author has written a book. We shall not make a critical analysis of this book, as the author has already done this so well that we see no reason for revising what he says on pages 17 and 18. Our advice is to get this book and memorize the author's analysis and then take time to study out the particular chapters in which you are specially interested. You will find all its chapters are so well written that you will not only be glad to have read them but you will want to own them for the information they contain.

We have rarely read a book on a subject in which we were not especially interested that has so charmed us. It may not be scientific for we cannot judge, but the editor and the publishers have produced a delightful book which we are glad to have, and to commend to every seeker for dental knowledge and real value.—Published by The C. V. Mosby Co., St. Louis, Mo.

